# BOO-ST and CBCEC: two novel hybrid machine learning methods aim to reduce the mortality of heart failure patients

**DOI:** 10.1038/s41598-023-48486-7

**Published:** 2023-12-18

**Authors:** Ananda Sutradhar, Mustahsin Al Rafi, F M Javed Mehedi Shamrat, Pronab Ghosh, Subrata Das, Md Anaytul Islam, Kawsar Ahmed, Xujuan Zhou, A. K. M. Azad, Salem A. Alyami, Mohammad Ali Moni

**Affiliations:** 1 Department of Computer Science and Engineering, Daffodil International University, Daffodil Smart City (DSC), Birulia, Savar, Dhaka, 1216 Bangladesh; 2 Department of Computer System and Technology, University of Malaya, 50603 Kuala Lumpur, Malaysia; 3 Department of Computer Science, Lakehead University, 955 Oliver Rd, Thunder Bay, ON P7B 5E1 Canada; 4 Department of Electrical and Computer Engineering, University of Saskatchewan, 57 Campus Drive, Saskatoon, SK S7N 5A9 Canada; 5 Department of Information and Communication Technology, Mawlana Bhashani Science and Technology University, Santosh, Tangail, 1902 Bangladesh; 6 Health Informatics Research Lab, Department of Computer Science and Engineering, Daffodil International University, Daffodil Smart City, Birulia, Dhaka, 1216 Bangladesh; 7 School of Business, University of Southern Queensland, Toowoomba, Australia; 8 Department of Mathematics and Statistics, Faculty of Science, Imam Mohammad Ibn Saud Islamic University (IMSIU), 13318 Riyadh, Saudi Arabia; 9 Centre for AI & Digital Health Technology, Artificial Intelligence & Cyber Future Institute, Charles Sturt University, Bathurst, NSW 2795 Australia

**Keywords:** Health care, Diagnosis

## Abstract

Heart failure (HF) is a leading cause of mortality worldwide. Machine learning (ML) approaches have shown potential as an early detection tool for improving patient outcomes. Enhancing the effectiveness and clinical applicability of the ML model necessitates training an efficient classifier with a diverse set of high-quality datasets. Hence, we proposed two novel hybrid ML methods ((a) consisting of Boosting, SMOTE, and Tomek links (*BOO-ST*); (b) combining the best-performing conventional classifier with ensemble classifiers (*CBCEC*)) to serve as an efficient early warning system for HF mortality. The *BOO-ST* was introduced to tackle the challenge of class imbalance, while *CBCEC* was responsible for training the processed and selected features derived from the Feature Importance (FI) and Information Gain (IG) feature selection techniques. We also conducted an explicit and intuitive comprehension to explore the impact of potential characteristics correlating with the fatality cases of HF. The experimental results demonstrated the proposed classifier *CBCEC* showcases a significant accuracy of 93.67% in terms of providing the early forecasting of HF mortality. Therefore, we can reveal that our proposed aspects (*BOO-ST* and *CBCEC*) can be able to play a crucial role in preventing the death rate of HF and reducing stress in the healthcare sector.

## Introduction

Heart failure (HF) is a complex and multifaceted medical condition that arises from the heart’s inability to meet the body’s metabolic demands. Despite considerable advancements in medical science, HF prevalence is still high and causes many deaths in industrialized and developing countries^1^. The most common causes of HF are sedentary behavior, excessive alcohol use, smoking, obesity, microbes, influenza, chest radiation, hypertension, cardiomyopathies, dyslipidemia, and so on^2^. Several non-lifestyle risk factors, including age, gender, family history, and high fibrinogen levels, could also be considered. Women^3^ and elderly persons^4^ are at a higher risk than men and younger people. Worldwide in 2018, a projected 64.3 million HF patients were estimated, with a total of 379,800 certified deaths^5^.

Examining the signs of mortality as soon as possible and beginning treatment with counseling and medications is crucial to reducing the fatality rate. Some conventional exploration like ejection fraction (measuring how well the heart pumps blood), B-type natriuretic peptide (a hormone released by the heart in response to HF), renal function (poor kidney function), and various clinical factors are examined to identify the risk of HF mortality. However, this manual process may not always be sufficient, and very complex, time-consuming, and expensive. As a result, researchers have concentrated on using machine learning (ML) methods to explore the signs of HF mortality.

Numerous studies have endeavored to explore a wide array of ML methods concerning these issues. However, these investigations have surfaced substantial challenges, leaving ample room for system enhancement. Likewise, the authors^6^ introduced bias and overfitting in the results section by integrating the imbalanced dataset into a predictive framework. Consequently, the studies^7–10^ have resorted to generating synthetic samples through the Synthetic Minority Oversampling Technique (SMOTE) and have thus prepared a balanced dataset prior to training. However, it is worth noting that SMOTE carries the risk of generating noisy and non-informative samples, which can potentially compromise the model’s efficiency^11^. To address these challenges, we introduce a novel method named *BOO-ST* that initially employs Boosting to pave the way for generating synthetic samples and enhancing the representativeness of the minority class^12^. Also, the Tomek link was considered to eliminate noisy and uninformative synthetic samples^13^. Through these strategies, we effectively mitigate existing issues and enhance the quality of minority instances, thereby reducing false positives and instilling greater confidence in critical condition predictions. Next, the authors^14,15^ have worked on a specific feature of the dataset without considering other potential characteristics of HF. Additionally, the studies^9,16^ utilized a feature selection technique and picked the training characteristics based on it. Nevertheless, without conducting a comparative evaluation of different feature sets, it is still questionable to incorporate features into a diagnostic model. Therefore, by using two robust feature selection techniques, Feature Importance (FI) by RF^8,17^ and Information Gain (IG)^9,10^, we make a comparative evaluation and aim to rectify the most potential characteristics of HF.

The preceding studies^7–9,14,16^ used single random sampling to validate the efficiency of their model, which can lead to biased results as the distribution of samples across classes did not accurately reflect the underlying population. To solve the issue, we have partitioned the training and validation data into multiple distinct subsets and evaluated the average results derived from these test splits. This approach provides a more dependable and precise assessment of the model’s performance. Subsequently, the studies^18–22^ have focused on conventional ML classifiers for the categorization of survival or death cases. However, conventional algorithms are susceptible to issues related to bias, over-fitting, and limited expressiveness^23^. The studies^8,24^ recommended a combination of multiple ML algorithms in the future to get multiple advantages at the same time and mitigate these drawbacks. Hence, the authors^25–27^ proposed some hybrid classifiers in their studies by using a single ensemble classifier. Nevertheless, still faced issues including limited diversity and overfitting associated with single ensemble classifiers^28^. In response to these concerns, we propose a novel classifier named *CBCEC*, by fitting our best-performing traditional classifier (BP-C) as the estimator of Bagging (BG) and leveraging another ensemble method Voting (VT). The BP-C can be eligible to lower the incorrect decisions and BG alleviates the overfitting issues during classification^29^. Moreover, combining two different ensemble methods (e.g., BG and VT) our proposed classifier can enhance the diversity in terms of the prediction and capturing of the complex data patterns. The incorporation of these capabilities into the proposed classifier enhances its predictive performance, adaptability, and robustness, thereby enabling it to handle a broader spectrum of ML tasks.

This research makes several contributions, including the introduction of a novel *BOO-ST* method to effectively overcome data imbalance issues and mitigate the issues related to SMOTE. Different feature sets are selected by performing two feature selection techniques (FI and IG) and picking the best one by evaluating multiple performance metrics. Then we utilized the fine-tuned parameters to control the learning process and conducted an ablution study for the proposed classifier *CBCEC*. A Partial Dependence Plot (PDP) is employed to identify the critical values range of HF mortality. Finally, the result section demonstrates the superiority of the proposed *CBCEC* classifier in terms of various predictive performances and statistical significance over the conventional and existing models.

## Related works

There have been several recent studies conducted on this topic. Most of the studies have focused on utilizing ML methods to detect the mortality of HF efficiently. For instance, Lili et al.^6^ aim to develop an ML-based predictive model for predicting the mortality risk of HF patients. Where the Xtreme Gradient Boost (XGB) classifier performed the highest results (82.4% area under the curve (AUC)) compared to others. Asif et al.^7^ have utilized some well-known ML classifiers (e.g., Random Forest (RF), AdaBoost (AB), K Nearest Neighbor (KNN), and Support Vector Machine (SVM)) to detect the mortality risk of HF. The result section demonstrates that RF performs better (76.25% accuracy) than other classifiers with chi-square-based selected features. ABID et al.^8^ attempted to find significant features using feature importance and mitigate the imbalance issue with SMOTE. From various classifiers, they identified ET outperforms with an accuracy of 92.62%. Saurav^9^ and Dafni et al.^10^ also attempted to overcome the imbalance issue by utilizing SMOTE. Then, the SVM and Rotation Forest Tree (ROT) classifiers performed the highest accuracy of 83.33% and 91.3%, respectively compared to others.

Chicco et al.^14^ aim to predict the survival of HF patients by employing only two characteristics of patients (e.g., serum creatinine and ejection fraction). Their predictive model gained an overall 74% accuracy from the RF classifier. After applying the grey wolf optimization feature selection method, Minh et al.^16^ compared the results of seven ML classifiers. From the result section, it is observed that RF generated the highest accuracy of 85%. Lal Hussain et al.^17^ employed various ML classifiers, where SVM obtained overall better performance with 88.79% accuracy with all multimodal features.

Mirza et al.^18^ utilized six conventional ML classifiers to analyze the UCI HF dataset. The RF classifier surpasses other classifiers with 90% accuracy when incorporating SMOTE-ENN and standard scaling. Prakash et al.^19^ attempted to predict the left ventricular ejection fraction changes in HF patients. Among the various prebuilt classifiers, XGB was identified as the highest-performing model with 88.6% AUC. Another study^20^ trained six supervised ML classifiers to build a model for predicting hospital mortality in HF. The authors claimed that RF gained the highest accuracy of 88% during the test phase. Employing the feature importance-based selected features, Sabahi^21^ and Cida^22^ obtained 76.4% accuracy and 83.1% AUC, respectively, using the XGB classifier.

A few researchers have presented some hybrid ensemble models in their studies. Such as, by combining the RF classifier with a linear model, Mohan et al.^24^ presented a hybrid model named HRFLM. Which has been found to produce a robust accuracy of 88.7%. Sohanur et al.^25^ proposed another hybrid model using Stacking (ST) with the integration of three conventional classifiers. Their proposed model outperformed the single prebuilt classifiers and achieved 89.41% accuracy. Pronab et al.^26^ presented some hybrid ensemble classifiers by the integration of single traditional classifiers. They have individually set the baseline classifier (e.g., RF, DT, AB, Gradient Boost (GB), and KNN) as a base estimator of Bagging (BG) and Boosting (BS). Another hybrid model was presented by Raza^27^ using an ensemble model named Voting (VT). Their proposed VT-based model outperformed conventional classifiers and demonstrated an effective accuracy of 88.88%.

## Research methodology

**Figure 1:**
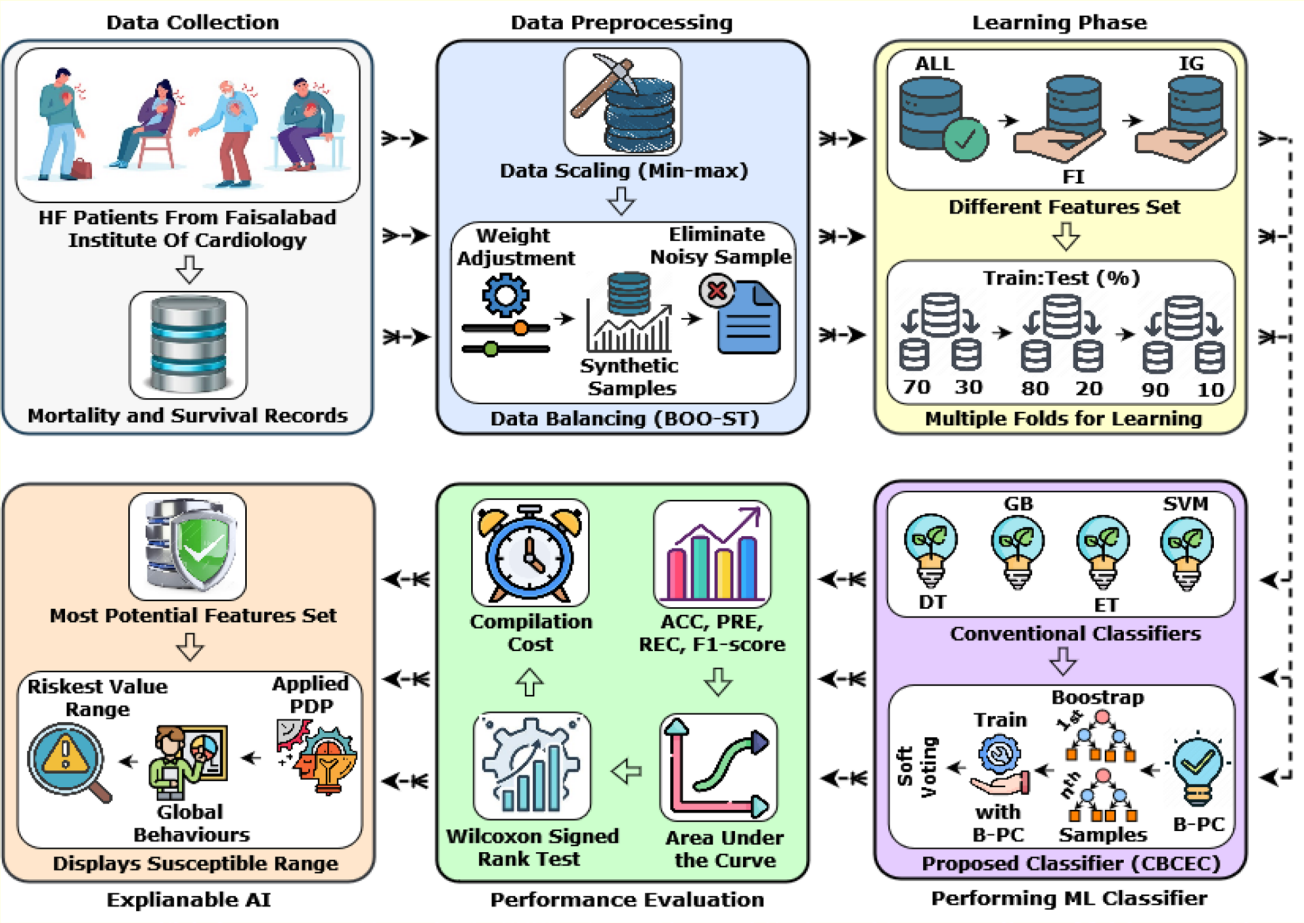
A schematic diagram highlighting the key methodologies of our study.

The current study uses numerous cutting-edge ML phases, such as preprocessing raw data, rectifying relevant features, classifying class levels, and exploring hidden factors. The raw data undergoes two critical preprocessing steps, namely data scaling, and balancing, which set the groundwork for downstream analysis. After that, the most significant features are handpicked using two widely accepted feature selection techniques, Feature Importance (FI) and Information Gain (IG). The training phase involves four conventional and a novel classifier proposed by us. To elucidate the complex interactions among the most preferred features, a Partial Dependence Plot (PDP) is employed to provide global explanations for each feature. Figure 1 illustrates the schematic diagram outlining the comprehensive workflow of our study.

### Data description

This study employed the Faisalabad Institute of Cardiology and Allied Hospital's heart failure clinical records dataset, which is now publicly available in the Kaggle data repository^30^. During the follow-up period from April to December 2015, 299 individual patients with heart problems—194 men and 105 women—made up the samples. Their age ranged between 40 and 95 years and all 299 patients had left ventricular systolic dysfunction and previous heart failures that placed them in the New York Heart Association (NYHA) categorization of heart failure stages III or IV. The average duration of the follow-up was 130 days, with a minimum of 4 days and a maximum of 285 days. Table 1 summarizes the employed dataset, including clinical, physical, and lifestyle features. Some features hold binary characteristics like Anaemia, High Blood pressure, Diabetes, Sex, Smoking, and DEATH_EVENT. The rest of them contain a mix of integer and float characteristics. Finally, for classification purposes, DEATH_EVENT has been selected as the target feature^7,8,14^, which states that if the patient died or survived (1 is for dead and 0 is for survived) before the conclusion of the follow-up period. Where 203 were dead and 96 surviving cases were reported.

**Table 1:** Dataset details with features explanation, measurement, and ranges of data.

Feature name	Explanation	Measurement	Range
Age	Patient age	Years	40–95
Anaemia	Decrease of red blood cells or hemoglobin	Boolean	0(no), 1(yes)
High blood pressure (H_b_p)	If the patient has blood pressure	Boolean	0(no), 1(yes)
Creatinine phosphokinase (Cr_ph)	Level of the CPK enzyme in the blood	Mgc/L	23–7861
Diabetes	If the patient has diabetes	Boolean	0(no), 1(yes)
Ejection fraction (Ej_fr)	Blood leaving percentage	Percentage	14–80
Sex	Man or woman	Binary	0(woman), 1(man)
Platelets	Platelets in the blood	Kilo platelets/mL	25.01–850.00
Serum creatinine (Se_cr)	Level of creatinine in the blood	mg/dL	0.50–9.40
Serum sodium (Se_so)	Level of sodium in the blood	mg/dL	114–148
Smoking	If patients smoke	Boolean	0 (no), 1(yes)
Time	Follow-up period	Days	4–285
DEATH_EVENT (target)	If the patient died in the follow-up period	Boolean	0(survived), 1(dead)

### Data preprocessing

The selected dataset for this study is almost clean and preprocessed; there are no missing values in this dataset. However, we consider two concerns that might prevent our model from getting a generalized outcome. For instance, there are huge differences between values in the case of creatinine phosphokinase and platelet features. It may delay the decision-making, hence overcoming this issue through min–max scaling. Which converts the feature values into a range; additionally, it helps quickly learn an algorithm and is essential for improving results.

#### Overcome the imbalance issue with *BOO-ST*

Nowadays, dataset imbalance is a common issue that mostly arises in publicly available datasets. It’s a situation when the number of instances in one class is significantly higher or lower than in another class. This can lead the model to bias toward the majority class, poor performance on the minority class, and misleading performance metrics. As a result, the researchers are quite concerned about this issue and seek to resolve it before training the data. The synthetic minority oversampling technique (SMOTE) is one of the famous approaches for balancing data and researchers mostly use it^7–10^. However, this strategy tends to produce noisy and irrelevant samples, while generating synthetic instances^11^.

In our study, we have addressed both imbalance and SMOTE-related issues by taking three crucial stages named *BOO-ST*. Typically, minority classes are frequently misclassified due to their underrepresentation and lack the sufficient examples to capture complex patterns. Therefore, at the initial step, we applied the boosting method on the imbalanced dataset $D$, over $T$ number of iterations. The dataset $D$ is trained on the equal weights $(1/n)$ of samples and calculates the learning rate $lr$, where $n$ is the total number of samples. Based on the learning rates, the weight is increased in the case of minority class samples. Resulting in the minority instances placing more emphasis on the next stages. Which is beneficial to improve the representation of the minority class and produce a more varied synthetic example^12^.

Following the weights adjustment of minority instances, we applied the SMOTE in the imbalanced dataset $\{(x1, y1), (x2, y2),\dots ,(xn, yn)\}$, where $xi$ is the feature vector of *ith* instances and $yi$ is the corresponding class level. Initially, it calculates the imbalance ratio by $\left|C\right| / |n|$, where $\left|C\right|$ and $|n|$ refer to the number of minority classes and the total number of samples respectively. Then calculates the k nearest neighbors $k(xi)$ from the minority classes $\left|C\right|$ and randomly selects the neighbors $xj$ from $k(xi)$. The difference between $xi$ and $xj$ for each feature dimension $d$ calculated using the formula $dif\left( v \right) = xi\_d {-} xj\_d$. After that, adding a fraction ($0<r<=1$) generates new synthetic instances $xs$, where $r$ is the random number between 0 and 1. Finally, newly generated synthetic instances $xs$ added to the augmented dataset $D{\prime}{\prime}$. Here, the potential noisy and irrelevant synthetic instances could make the model prone to high complexity and difficulty reproducing results. Hence, in the final stages, we try to eliminate these drawbacks from our study and apply Tomek links to the augmented dataset $D{\prime}{\prime}$. In the Tomek link procedure, we again determine k nearest neighbors from both minority and majority samples from $D{\prime}{\prime}$, denoted as $k(xk)$ and $k(xkd),$ respectively. This step entails computing the Euclidean distance between $xi$ and all instances of $D{\prime\prime}$’ and selecting the $p$ instances from both classes with the smallest distances. Afterwards, locate the desired samples of the majority class data that are closest to the minority class data (i.e., the majority class data that makes the minority class data distinct from ambiguous) and then remove it. Following these procedures, we can greatly reduce the complexity of $D{\prime}{\prime}$, by removing noisy and irrelevant samples^13^. The proposed *BOO-ST* method significantly generates 198 of the total samples in the survival class. The whole working process of the *BOO-ST* is illustrated in Algorithm 1.

**Algorithm 1:**
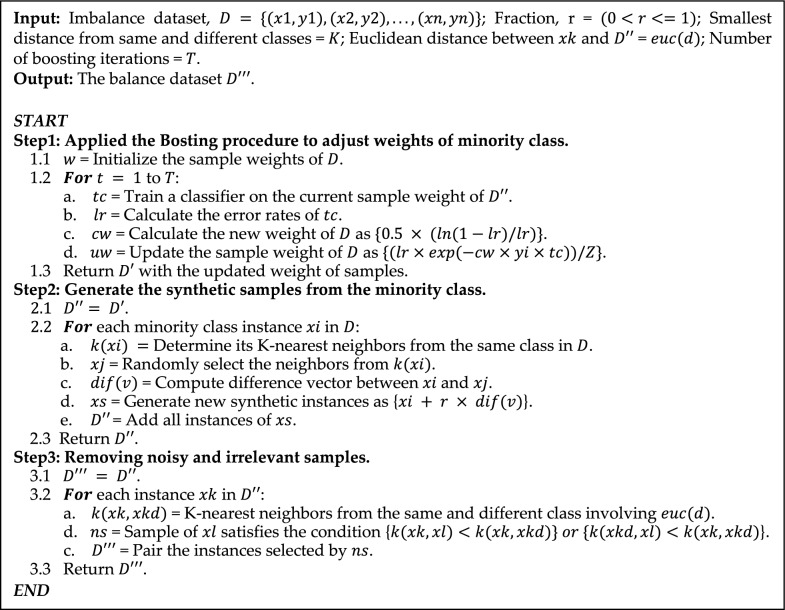
Illustrates the procedures of a novel data balancing method, BOO-ST, consisting of multiple effective machine learning strategies.

### Feature selection and learning phase

Feature selection is a pivotal technique that significantly refines machine learning performance by identifying the most critical variables and discarding the insignificant ones. To improve the overall efficiency of the process, the present study employs two effective feature selection techniques, namely feature importance (FI) and information gain (IG). FI assigns a score to each input feature based on its importance in predicting the outcome of interest, thereby offering insights into the contribution of each variable towards the model and its prediction accuracy. A Random Forest is fitted with the FI method to rank the features. On the other hand, IG is an entropy-based feature selection approach that measures the gain of each variable concerning the target variable. It focuses on identifying how much information a phrase can be used to categorize. After conducting these feature selection methods, the top ten most significant features are selected based on their importance rank, Table 2 states these features with ranks. The processed dataset and the reduced feature sets are divided into 70, 80, and 90% for the training and, in response, 30, 20, and 10% for testing respectively. Further, averaging the obtained results from multiple testing splits to validate the model performance. This can provide a more reliable and robust assessment of model performance.

**Table 2:** Rectify the most significant features of heart failure from two feature selection methods: feature importance-based selected features, and information gain-based selected features.

Feature importance by RF		Information gain	
Selected features	Importance rank	Selected features	Importance rank
Time	0.36	Time	0.33
Se_cr	0.26	Ej_fa	0.24
Ej_fa	0.21	Se_cr	0.20
Age	0.17	Age	0.14
Cr_ph	0.15	Anaemia	0.11
Plateletes	0.12	Cr_ph	0.08
Se_so	0.10	Se_so	0.07
Sex	0.10	Plateletes	0.05
Diabetes	0.08	Diabetes	0.05
Smoking	0.07	H_b_p	0.03

### Classifiers description

In our quest to identify HF, utilized four well-established machine learning classifiers: decision tree, gradient boost, support vector machine, and extra tree. In addition, to improve classification performance, we have also proposed a novel combinational ML classifier, named *CBCEC*. A detailed description of the performed classifiers is provided in the following subsections.

#### Decision tree

The way a decision tree (DT) operates is by iteratively segmenting the input data into subsets according to the value of one of its attributes. Regarding the target variable, the subsets are partitioned in a way that makes them as homogeneous as possible. The highest information gain (IG) is chosen as the feature to use for this, which is stated in Eq. (1). The result is a tree-like structure where each leaf node represents a class label, and each inside node represents a test on a feature.

1 $$IG\left({D}_{p},f\right)= I\left({D}_{p}\right)- {\sum }_{j=1}^{m}\frac{{N}_{j}}{{N}_{p}}I\left({D}_{j}\right)$$

where $f$ is the feature on the dataset is ${D}_{p}, I({D}_{p})$ is the impurity of dataset ${D}_{p},$ ${N}_{p}$ is the total number of instances in ${D}_{p}$, ${N}_{j}$ is the number of instances in subset ${D}_{j},$ and $I({D}_{j})$ is the impurity of subset ${D}_{j}.$

#### Gradient boost

Gradient Boost (GB) is an ensemble ML approach that generates predictions using a few decision trees. It functions by adding new decision trees in a sequential manner to fix errors in the preceding trees, hence reducing the overall error. The combined forecasts of all the trees are weighted to provide the final prediction, evaluated in Eq. (2).

2 $$y\left(x\right)=F\left(x\right)+ \sum_{i}{h}_{i}(x)$$

where $y(x)$ is the predicted output, $F(x)$ is the initial model prediction, $\sum_{i}{h}_{i}(x)$ is the sum of the predictions of all the decision trees, ${h}_{i}\left(x\right)$ is the prediction of the ${i}^{th}$ decision tree, which is trained to correct the errors of the ${(i-1)}^{th}$ tree.

#### Support vector machine

Support Vector Machine (SVM) is a potent supervised learning method that may be used for regression and classification. To separate the various classes in the dataset, SVM searches for the optimal decision boundary or hyperplane^31^. The basic goal is to choose a hyperplane with the greatest margin—that is, the distance between the hyperplane and the closest data point for each class. The working function of SVM is illustrated in Eq. (3).

3 $$S\left(x\right)=sign({w}^{T}x+b)$$

where $x$ represents the input data, $w$ represents the weight vector, $b$ is the bias term, $T$ denotes the transpose, and $sign()$ is a sign function that, depending on the type of input data, returns either $+1$ or $-1$.

#### Extra tree

An Extra Trees Classifier (ET) is an ensemble learning approach that randomly constructs numerous decision trees and integrates their outputs to increase the model's overall accuracy. In ET, a random split point is selected rather than looking for the best split point in the feature space as in conventional decision trees. A vast number of decision trees are constructed using this method, each of which has a random split point for each feature. The mathematical procedures are represented in Eq. (4).

4 $$E\left(y\right)={\sum }_{i=0}^{n}{\mathrm{w}}_{\mathrm{i}}{h}_{i}(x)$$

where $E(y)$ refers to the predicted outcome, $n$ refers to the total number of decision trees, ${w}_{i}$, and ${h}_{i}$ are the weight and predicted output of ${i}^{th}$ tree respectively for the input $x$.

#### Combining the best-performing conventional classifier with ensemble classifiers

In the realm of ML, the development of effective predictive models is paramount, yet conventional ML classifiers often grapple with issues of bias, overfitting, and limited generalization^23^. Hence, recently numerous studies^25–27,32,33^ have attempted to introduce hybrid ensemble models to solve the difficulties efficiently. Recognizing the limitations of conventional ML and single ensemble method (limited diversity and overfitting^28^), this study introduces a novel approach named *CBCEC* by harnessing the power of hybrid ML classifiers, which seamlessly blend the strengths of different algorithms to enhance prediction accuracy, model robustness, and adaptability. The novel classifier *CBCEC* is developed by combining one general and two ensemble classifiers, Bagging (BG), and Voting (VT). BG is a kind of ensemble ML method that mixes the results of numerous learners to enhance performance. It mainly works on bootstrapping (creating some bootstrap data samples from the data) and aggregating (aggregating the individual predictions from each bootstrap sample). The primary job of VT is to integrate the predictions of various independent classifiers and forecast the class that will receive the most votes or probabilities. It can enhance the model's overall accuracy and resilience by lowering variance and bias.

Different classifiers have different strengths and weaknesses, which can vary on the datasets. Choosing the wrong classifier in the hybrid combinational method can lead to poor performance, incorrect predictions, and decisions. Whereas the preferred one can significantly impact the accuracy and reliability of the predictions. Hence, we initially trained four traditional classifiers and determined the best-performing classifier ($BP-C$) by comparing the performed results. Evaluated in Eq. (5), where ${D}_{test}$ is the test instances for each classifier and $Ma{x}_{ACC}$ refers to the maximum accuracy from the test phase.

5 $$B-PC = Ma{x}_{ACC}\{DT\left({D}_{test}\right), GB({D}_{test}), SVM\left({D}_{test}\right), ET({D}_{test})\}$$

Then set $B-PC$ as a base estimator and parallelly fit for training the generated bootstrap samples of BG, let as $B-BG$. In Eq. (6), ${D}_{b}$ and ${D}_{B}$ are the first and last bootstrap samples, respectively. Training all the bootstrap samples helps to capture the underlying patterns and relationships of the dataset. Finally, aggregate the predictions from all bootstrap samples ${D}_{b}$ to ${D}_{B}$ and reduce the chances of overfitting^29^. Additionally, it could be superior in reducing variance without making biased results.

6 $$B-BG={\sum }_{b=1}^{B}\{B-PC\left({D}_{b}\right),\dots \dots .,B-PC\left({D}_{B}\right)\}/B$$

Another ensemble classifier VT can perform well when two or more base classifiers fit together^34^. Hence, we finally integrate $B-PC$ and $B-BG$ using the soft voting. This type of voting works with multiple classifiers and generates the average probability score for all classes; finally, the highest average prediction is selected to create the final prediction, as stated in Eq. (7). Which can enhance the confidence or certainty of the model predictions. Furthermore, by combining the prediction of multiple classifiers with different biases and error rates, *CBCEC* can reduce the overall biases and errors in final predictions. Algorithm 2 holds the whole procedure of $CBCEC$ the classifier.

7 $$CBCEC=agrmax\{B-PC\left({D}_{train}\right), B-BG\left({D}_{train}\right)\}$$

**Algorithm 2:**
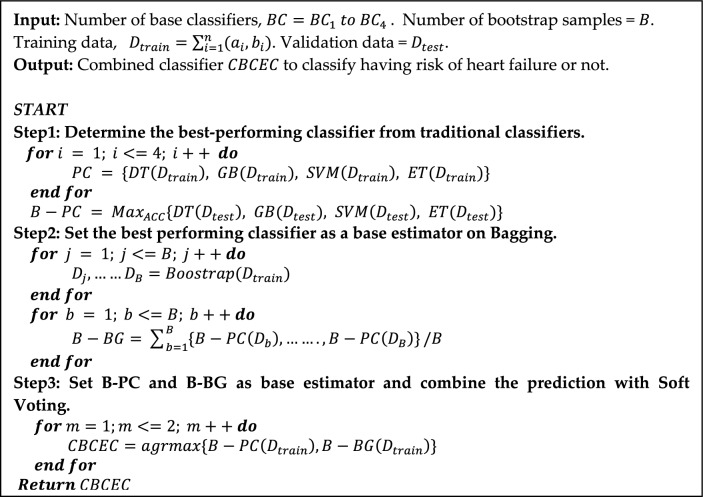
Develop a novel hybrid machine learning classifier by combining best-performing conventional classifiers and two robust ensemble methods to detect heart failure mortality efficiently.

### Ablation study of the proposed classifier

Before embarking on the journey of model development, it is essential to lay a solid foundation. This is precisely what our ablution study accomplishes. This study serves as the critical groundwork for ensuring the feasibility, viability, and ultimate success of our model. Three distinct experiments were undertaken through this study (e.g., the base estimator, random state, and voting type), wherein various facets of the proposed *CBCEC* classifier were systematically modified. This rigorous examination of different components aimed to cultivate a more robust architecture, ultimately resulting in heightened classification accuracy.

#### Experiment 1: modification of base estimators

The base estimator refers to the individual ML classifiers that make up the ensemble or hybrid model. Fitting an appropriate base estimator is crucial for the hybrid ensemble method, as it directly influences the overall performance, robustness, and ability to provide accurate predictions across diverse scenarios. Hence, we individually fit each conventional classifier as a base estimator on both ensemble methods (BG and VT) and obtained the performances. Table 3 shows the outcomes for each case, where the GB produces 93.67% accuracy for FI features set as a base estimator and performs slightly better compared to others.

**Table 3:** Modification of the base estimators to conduct an ablation study, where the sign (✓) and (✘) refer to the identical and dropped accuracy, respectively.

Case study	Base estimator	ALL features	FI features	IG features	Acceptability
1	DT	88.75	92.5	92.5	✘
	GB	89.74	93.67	92.40	✓
	SVM	87.5	90	88.75	✘
	ET	90	92.5	91.25	✘

#### Experiment 2: modification of random states

The random state is used as a parameter of the ML model that controls the randomness or unpredictability of certain operations. Selecting appropriate random states enhances the reliability, reproducibility, and fairness of our proposed classifier. It ensures that the results are not influenced by random variations. To identify the ideal state of random we conduct a comprehensive evaluation of different numbers of states. As shown in Table 4, when specifying the random state as 10 our proposed classifier demonstrated an identical score of 93.67% accuracy, which is close to the random state of 15 and 25.

**Table 4:** Modification of the random state to conduct an ablation study, where the sign (✓) and (✘) refer to the identical and dropped accuracy, respectively.

Case study	Random state	ALL features	FI features	IG features	Acceptability
2	5	88.9	92.5	90.12	✘
	10	89.74	93.67	92.40	✓
	15	88.75	92.59	88.75	✘
	20	88.9	91.25	90	✘
	25	88.75	92.5	91.25	✘
	30	90	91.25	92.59	✘
	35	88.75	91.25	90	✘
	40	89.74	90	89.74	✘

#### Experiment 3: modification of the voting types

There are three different VT schemes in ML, these have different behaviors and can lead to variations in the model performance. The choice of VT type can significantly influence the overall performance as it tailors the model’s behavior to the specific requirements of the problem. Table 5 illustrates the performance of our proposed classifier using three different VT types (e.g., hard, weighted, soft). The table reveals that the soft VT produces the maximum test accuracy compared to hard and weighted. Therefore, we have selected the soft VT for further exploration of our proposed classifier.

**Table 5:** Modification of the voting type to conduct an ablation study, where the sign (✓) and (✘) refer to the identical and dropped accuracy, respectively.

Case study	Voting type	ALL features	FI features	IG features	Acceptability
3	Hard	89.74	92.5	92.40	✘
	Weighted	90	92.59	91.25	✘
	Soft	89.74	93.67	92.40	✓

## Experiments and results

This section comprehensively evaluates the experimental results obtained from our proposed methodology. To ensure a thorough analysis, we have measured various classification metrics of both traditional and proposed classifiers for all three scenarios (e.g., All features, FI-based features, and IG-based features). Then explore the global behaviors from the most potential features selected from this comparison.

### Experimental setup

The efficiency of the proposed and baseline classifiers was evaluated through modeling experiments using computer equipment with an Intel Core $i3$ processor of 10*th* GEN clocked at 3.3 GHz and 4 GB of RAM. The cloud-based Jupyter Notebook environment (Colab NoteBook) was used for constructing and prototyping the performed methods. Since it has several freely available suitable libraries for ML models (e.g., *Scikit-learn*, *Mathplotlib*, *Keras*, and so on).

### Evaluation metrics

Several evaluation metrics, namely accuracy, precision, recall, f1-score, an area under the curve (AUC), and computational cost measured to show the robustness of our research in terms of classification^35^. Accuracy quantifies the percentage of accurate classifications the model makes. Recall measures the model's ability to recognize positive instances accurately and precision measures the model's capacity to produce accurate positive predictions. A balanced indicator of the model's overall performance, the F1-score combines precision and recall. The strategy of accuracy, precision, recall, and f1-score are stated in Eqs. (8–11). Where $TP$, $FP$, $FN$, and $TN$ refer to the number of true positives, the number of false positives, the number of false negatives, and the number of true negatives, respectively^36^.

8 $$Accuracy = TP+TN / (TP+FP+TN+FN)$$

9 $$Precision = TP / (TP+FP)$$

10 $$Recall = TP / (TP + FN)$$

11 $$F1-score = (2*Precision*Recall) / (Precision+Recall)$$

The AUC is an essential evaluation statistic that gauges the level of separability between the two classes. Additionally, compilation complexity gains insight into the computational performance of the employed classifiers. Furthermore, to evaluate the statistical significance of the proposed classifier over various feature sets, we conducted a statistical hypothesis test named the Wilcoxon signed rank test*.*

### Analysis of the performed result

On three different feature sets, we thoroughly compared the proposed *CBCEC* classifier to four conventional classifiers, DT, GB, SVM, and ET. The entire comparison enabled us to identify the most essential features for predicting HF mortality and assess the effectiveness of the proposed *CBCEC* classifier in comparison to the traditional classifiers. A thorough summary of the comparison's results is provided in the ensuing subsections.

#### Evaluation of the accuracy, precision, recall, and F1-score

**Figure 2:**
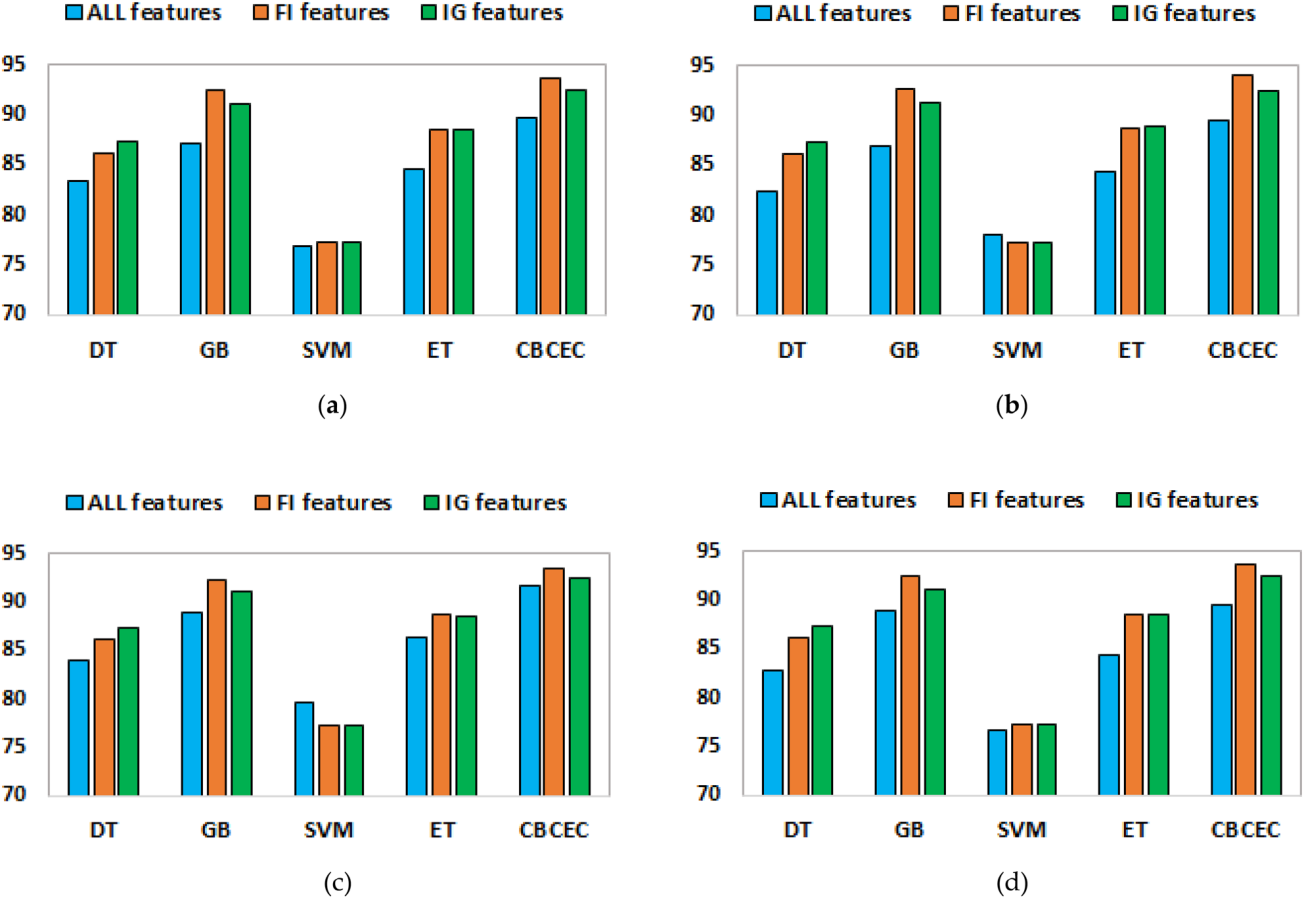
A comparative analysis between the traditional and our proposed classifier over three different features set based on some performance matrices of (**a**) accuracy, (**b**) precision, (**c**) recall, and (**d**) F1-score.

Figure 2a illustrates the accuracy of all classifiers for three distinct feature sets. Notably, the proposed classifier *CBCEC* emerges as the top performer with a remarkable accuracy rate of 93.67% with the FI-based features set. While the SVM classifier achieved a mortality detection rate of 77.21%, which was relatively consistent across other feature sets. As opposed to the baseline classifiers, the GB classifier excels by reaching an accuracy rate of 91.92% for the identical feature set. Then the precision score of Fig. 2b, also reveals that the *CBCEC* achieved the highest precision scores of 92.57% and 94.02% when trained with the IG and FI-based reduced features sets, respectively. It is worth mentioning that SVM performed the lowest precision scores, ranging from 77 to 78%, for all different feature sets.

According to Fig. 2c, once again *CBCEC* achieved a strong result as a recall score of 93.51%, whereas SVM obtained the lowest recall score of 77.18% with the FI features. Finally, the results of f1-scores from the classifiers are displayed in Fig. 2d. Interestingly, the DT, GB, ET, and *CBCEC* yielded f1-scores within the 80% to 94% range for all different feature sets. It is worth noting that the *CBCEC* using the FI-based feature set obtained the highest f1-score of 93.63%. Overall, we can demonstrate that the *CBCEC* consistently performs well across various evaluation metrics.

#### Performance analysis based on the area under the ROC curve

**Figure 3:**
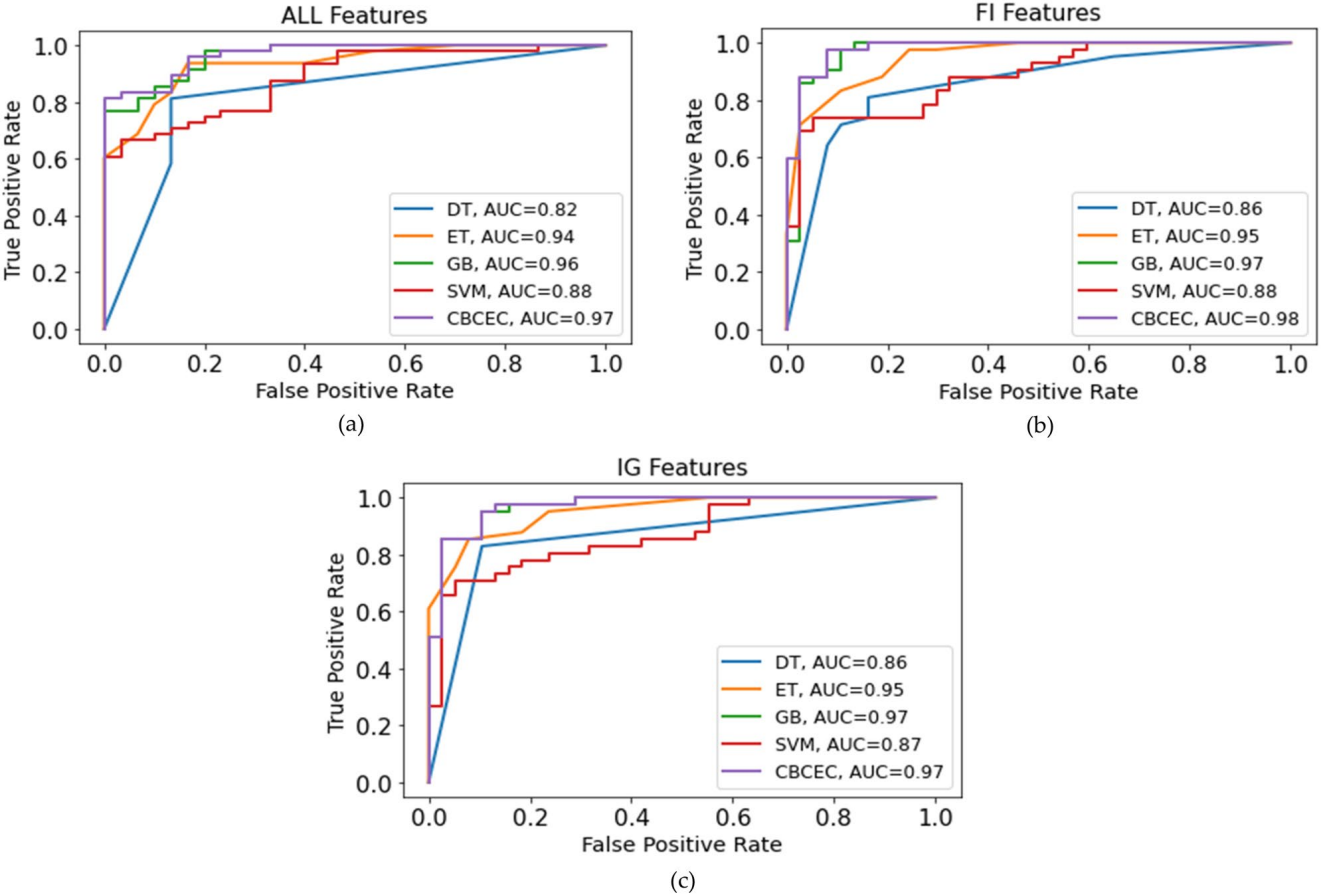
Analysis of the AUC scores of the performing algorithms on the three different feature sets, (**a**) all features, (**b**) FI features, and (**c**) IG features.

Figure 3 illustrates the area under the curve (AUC) of all classifiers implemented on three different feature sets, i.e., ALL Features (a), FI Features (b), and IG Features (c). Where, the x and y-axis represent the false positive and true positive rates, respectively, and the AUC scores of each classifier are depicted on the label. It can be observed that the *CBCEC* has produced the highest AUC score of 98% with the FI-based selected features. This result indicates that the proposed classifier is proficient in distinguishing between the two classes, making it a reliable model for predicting HF.

#### Computational complexity

Measuring computational complexity is a fundamental aspect of developing an ML model. It guides the optimization of the proposed classifier and ensures practical feasibility for the given task within the available resources. To gain insight into the computational performance, we carefully reported the respective execution time in milliseconds (MS) and required space in bytes (BT) for all performing classifiers, displayed in Table 6. Interestingly, the proposed *CBCEC* showed a comparatively higher runtime, approximately 1351, 957, and 754 MS for all, FI, and IG-based features, respectively. As it needs to undertake multiple steps during the execution. Additionally, this classifier demands high network spaces, for example, 2,476,100, 2,471,340, and 2,475,788 BT for ALL, FI, and IG features, respectively. At the same time, DT was found to have the lowest time (15.3, 12.2, and 11.8 MS) and space (7145, 7097, and 7113 BT) compared to others. These findings significantly emphasize the need for future research to create classifiers that can provide high performance while keeping computational costs low.

**Table 6:** Computes the time and space complexity in MS and BT, respectively for each classifier based on the different feature sets.

Features set	Time complexity					Space complexity				
	DT	GB	SVM	ET	CBCEC	DT	GB	SVM	ET	CBCEC
ALL	15.3	106	82.8	53.2	1351	7145	172,333	38,555	1,807,929	2,476,100
FI	12.2	105	77.1	26.3	957	7097	172,301	33,499	1,720,345	2,471,340
IG	11.8	82.2	53.6	24.5	754	7113	170,140	33,515	1,740,521	2,475,788

#### Wilcoxon’s signed rank test

The Wilcoxon signed rank test (WSRT)^37^ is a statistical hypothesis test that is used to compare several samples and classifiers. Using WSRT, it can determine whether there is a substantial difference between the paired classifiers with samples. Here we measure the test statistics (TS) and *P*-values using WSRT for the possible pairs of all classifiers based on the accuracy. To calculate the test statistic (TS), the differences between the matched measurements are ranked summarily. Besides that, the *P*-value is calculated by comparing the TS to a critical value or approximation based on the normal distribution. It is possible to reject the null hypothesis in favor of the alternative hypothesis, which is that there is a difference between the paired measurements if the p-value is smaller than the selected significance level (0.05). Table 7 shows that our proposed classifier *CBCEC* generates the TS value 2.0 up to 70.0 by pairing other classifiers for all different feature sets. It means that the sum of the ranks of the positive differences or the negative differences is equal to 2.0–70. This value represents how much the two samples under comparison in the test differ from one another. In the case of *P*-value, we see that most of the paired groups of classifiers (e.g., DT vs. GB, DT vs. SVM, DT vs. *CBCEC*, GB vs. *CBCEC*, SVM vs. *CBCEC*) have lower scores for three different feature sets, like less than the threshold or significant level of 0.05. This indicates that the differences between the paired classifiers, particularly the proposed *CBCEC* classifier is statistically significant for all different feature sets.

**Table 7:** Displays the test statistic (TS) and *P*-value for all possible pairs of different classifiers on three feature sets (ALL, FI, and IG-based features) based on the accuracy of each classifier, where the significant level (SL) is set as 0.05.

All possible pairs of employed classifiers	ALL features (SL = 0.05)		FI features (SL = 0.05)		IG features (SL = 0.05)	
	TS	*P*-value	TS	*P*-value	TS	*P*-value
DT versus GB	4.5	0.03389	6.0	0.06572	5.0	0.04523
DT versus SVM	25.5	0.01241	88.0	0.27523	66.5	0.34577
DT versus ET	28.0	0.16551	22.0	0.52708	10.5	0.69745
DT versus CBCEC	4.5	0.02389	10.5	0.06734	2.0	0.56370
GB versus SVM	37.5	0.28504	51.0	0.31731	84.0	0.37109
GB versus ET	20.0	0.73888	8.0	0.25683	18.0	0.45674
GB versus CBCEC	3.0	0.03256	1.0	0.04131	2.0	0.04131
SVM versus ET	28.0	0.16551	45.0	0.08955	51.0	0.31731
SVM versus CBCEC	37.5	0.02504	40.0	0.01967	70.0	0.02134
ET versus CBCEC	20.0	0.07388	7.0	0.41421	12.0	0.07045

### Global behaviors of the most impactful features

**Figure 4:**
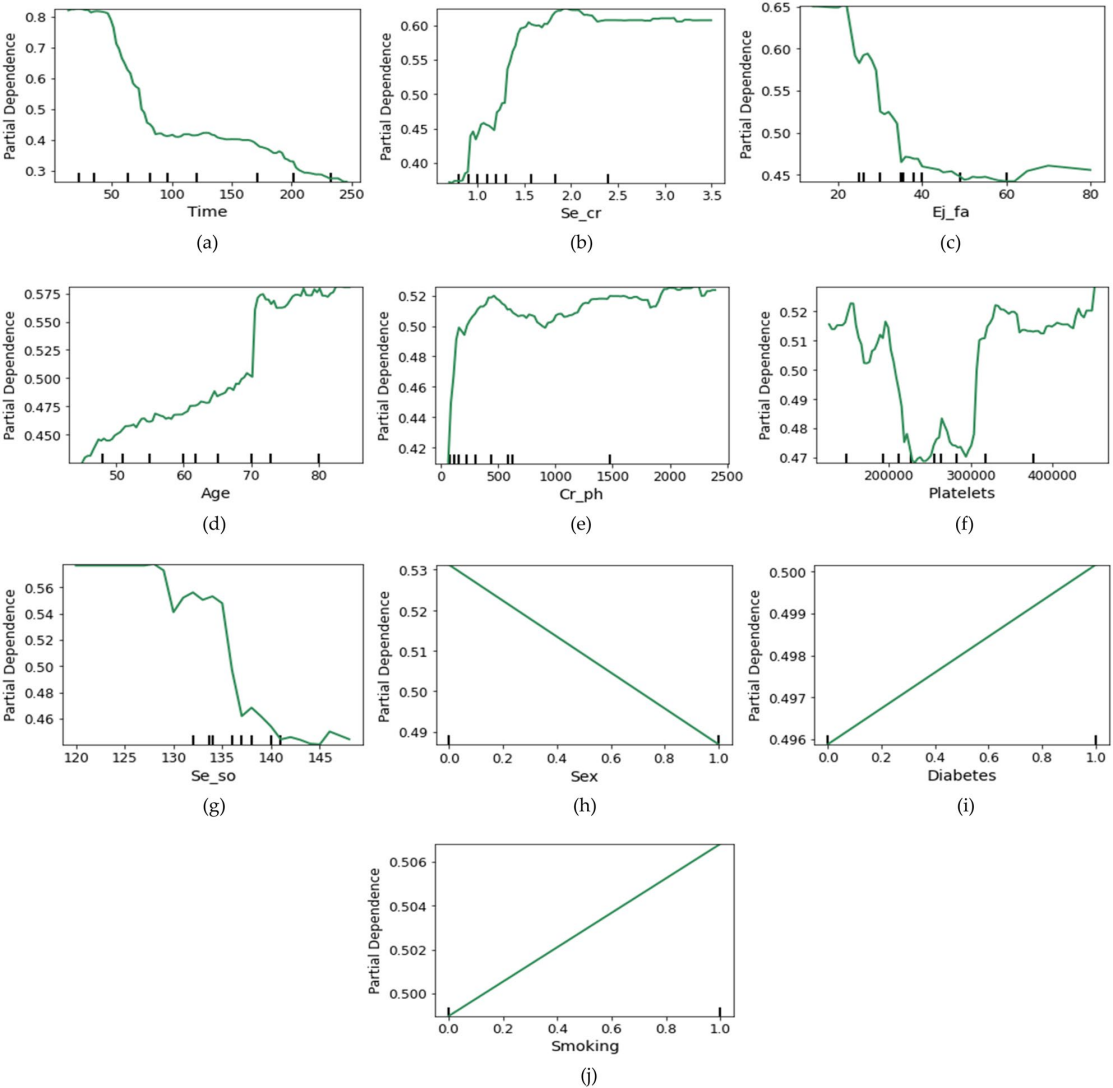
Presented the partial dependence plot (PDP) for the most impactful features (e.g., (**a**) time, (**b**) serum creatinine, (**c**) ejection fraction, (**d**) age, (**e**) creatinine phosphokinase, (**f**) platelets, (**g**) serum sodium, (**h**) sex, (**i**) diabetes, (**j**) smoking) of our findings.

Enhancing the interpretability and transparency of ML models explainable AI (EAI) enables stakeholders to understand the hidden process. This is the most practical way to increase patient care and safety by offering hidden explanations, especially in the medical field. Hence, we have utilized an EAI method named Partial Dependence Plot (PDP) to generate global behaviors for the most potential features (FI features) of HF. The function of a PDP is to visualize the relationship between a selected feature and the outcome predicted by a ML model while keeping other features constant. It computes the average expected outcome for the chosen feature over a range of values and then graphs these average forecasts against the feature values. Which enables us to determine whether there are any nonlinear or interactional effects and how the feature affects the model's anticipated result. Figure 4 illustrates the PDP plot for the FI-based features, where the y-axis represents the partial dependence of the feature, and the x-axis holds the feature's value. The minor ticks on the x-axis depict the various values of the features and the color line (lime) is the PDP line. When this line is relatively high for the specific feature values, it indicates this value range is susceptible to HF mortality.

The generated PDP plots help us interpret and identify the riskiest value ranges or classes of each feature, raising awareness among stakeholders and patients. To provide more clarity, we summarize the riskiest value ranges or classes for each feature in Table 8. Additionally, gather the existing explanations for all characteristics, which can validate the effectiveness of our findings. From this table, the stakeholders and patients will discover what possible value ranges or classes could result in HF-related death.

**Table 8:** The riskiest heart failure value ranges are determined using the interpretable partial dependence plot (PDP) for the most significant characteristics of our findings.

Feature	Susceptible value range or classes	Existing justification
Time	Within 4–40 follow-up days	Recommended follow-up within 14 days^38^
Se_cr	Within 1.5–3.5 mg/dl	A higher Se_cr value can increase mortality^39^
Ej_fa	Within 14–20 percent	Below 30% is severely abnormal Ej_fa^40^
Age	Within 70–95 years	HF mostly occurs in older people^41^
Cr_ph	Within 200–2500 mcg/L	10–120 mcg/L is normal, otherwise abnormal^42^
Platelets	< 100,000 and > 350,000 per uL	Moderate to severe platelets < 100,000 per uL^43^
Se_so	Within 114–130 mEq/L	< 135 mEq/L is the prevalence value of Se_so in HF^44^
Sex	Women	Women are more prone than men to suffer from HF^45^
Diabetics	Having diabetics	People with diabetes are more susceptible to HF^46^
Smoking	If smoke	Smoking can cause HF^47^

## Discussion

The rising demand for high-quality healthcare services has made machine learning methods essential for the medical industry. Through the automation and improvement of numerous healthcare procedures, including detection, diagnosis, treatment, and monitoring, these techniques have the potential to reduce the stress of healthcare personnel significantly. Hence, we develop an effective system for detecting HF mortality by two novel ML methods named *BOO-ST* and *CBCEC*.

Initially, instead of employing the conventional methods, we have presented a novel technique called *BOO-ST* to address the imbalanced problem of the dataset. This strategy enhances the quality of synthetic minority instances by emphasizing their weights through several iterations. After successfully completing each iteration, it eliminates noisy and irrelevant synthetic instances to help the model focus on the informative patterns. The proposed *BOO-ST* is a powerful technique for addressing the imbalance issue and improving the fairness of ML models, especially in situations where minority class detection is of utmost importance. Following the robust feature selection techniques FI and IG, the detection phase involved the implementation of four traditional and one proposed classifier *CBCEC*. To reduce the misclassification rate, it was developed by combining the best-performing conventional classifier. According to the earlier section, GB was identified as the top-performing classifier since it outperformed the four baseline classifiers, and we incorporated it with other ensemble classifiers. Notably, we found that FI-based selected features yielded superior results compared to ALL and IG features. Thus, we can confidently state that FI-selected features have a more significant impact on the overall accuracy of our proposed classifier. However, the model’s generalizability could be affected by unusual data conditions, which may cause overfitting and underfitting during classification.

To mitigate these issues, the training data was cleaned and preprocessed by *BOO-ST*. By generating diverse synthetic samples, this proposed strategy helps to reduce overfitting and underfitting^12^. Additionally, the *CBCEC* classifier was developed by combining multiple ensemble classifiers, which would be grateful to reduce these issues^28^. Then we control our learning process utilizing hyperparameter tuning and ablation study, which potentially reduce the model complexity and overfitting issues. Therefore, we can hypothesize that our proposed system is less prone to these issues and produces a highly generalized model. Moreover, a comparison summary based on the outcomes of our proposed aspects and state-of-the-art has been presented in Table 9. Which could be beneficial for further investigations and provide a fresh perspective on the topic. The table shows that our proposed aspects (*BOO-ST* and *CBCEC*) are more generalized and accurate than previous studies producing an accuracy of 93.67%.

**Table 9:** A direct comparison between the existing studies and our findings is based on the performance results, where the short form of ACC, AUC, and TC refers to accuracy, area under the ROC curve, and time complexity, respectively.

Year and reference	Data collection Source	Number of instances	Type of target class	Reduce imbalance issues	The performing classifiers	Best performingclassifier	The performed results
2022 ^6^	The eICU-CRD (version 2.0)	2798	Binary	–	XGB, LR, RF, SVM	XGB	ACC = 82.6%, TC = –
2021 ^7^	Faisalabad Institute of Cardiology	299	Binary	SMOTE	RF, AB, KNN, SVM	RF	ACC = 76.25%, TC = –
2021 ^8^	Faisalabad Institute of Cardiology	299	Binary	SMOTE	DT, RF, ET, SVM, GB	ET	ACC = 92.62%, TC = –
2022 ^9^	Faisalabad Institute of Cardiology	299	Binary	SMOTE	SVM, DT, RF	SVM	ACC = 83.33%, TC = –
2021 ^10^	Ireland and University Hospital of Ioannina	487	Multiple	SMOTE	DT, RF, KNN, SVM, LMT, ROT	ROT	ACC = 91.23%, TC = –
2020 ^14^	Faisalabad Institute of Cardiology	299	Binary	–	RF, DT, GB, LR, SVM, KNN, NB	RF	ACC = 74%, TC = –
2021 ^16^	The University of California Irvine	299	Binary	–	DT, SVM, KNN, RF	RF	ACC = 87%, TC = –
2021 ^17^	Physionet databases	NA	Multiple	–	DT, SVM	SVM	ACC = 88.79%, TC = –
2022 ^18^	Faisalabad Institute of Cardiology	299	Binary	SMOTE-ENN	RF, DT, SVM, KNN, LR	RF	ACC = 90%, TC = –
2023 ^20^	PMRCardio database	500	Binary	–	RF, LR, SVM, GB, XGB	RF	ACC = 88%, TC = –
2023 ^21^	Persian Registry Of cardio Vascular diseasE	2918	Binary	Undersampling	DT, RF, XGB LR, SVM, KNN	XGB	ACC = 76.4%, TC = –
2022 ^22^	Medical Information Mart for Intensive Care	46,520	Binary	–	XGB	XGB	AUC = 83.1%, TC = –
2019 ^24^	The University of California Irvine	303	Binary	–	DT, RF, SVM, GB, HRFLM	HRFLM	ACC = 88.7%, TC = –
2023 ^25^	Physionet	2008	Binary	–	XGB, RF, ET, GB, SVM, KNN, ST	ST	ACC = 89.41%, TC = –
2019 ^27^	The University of California Irvine	270	Binary	–	LR, NB, MLP, VT	VT	ACC = 88.88%, TC = –
2023 Our Study	Faisalabad Institute of Cardiology	299	Binary	BOO-ST	DT, SVM, ET, KNN, CBCEC	CBCEC	ACC = 93.67%, TC = 957 ms

The signs (–) indicate that the existing studies did not consider specific performance metrics or methods in their model.

## Conclusions

Despite significant medical improvements, clinicians find it more difficult to reduce the prevalence of heart failure mortality. Hence, this study aimed to develop an ML-based early warning system to detect mortality due to heart failure. To achieve this goal, initially, we overcome the difficulties of imbalanced data with a novel combined method named *BOO-ST* and rectify the potential features followed by two robust feature selection methods. Experimental results demonstrated that the proposed *CBCEC* classifier has a significant ability to detect mortality with Feature Importance (FI)-based selected features. Moreover, exploration of the susceptible value ranges of HF mortality could help patients understand their conditions and take appropriate actions. We believe that our proposed approach has the potential to advance the medical field and benefit HF patients by providing early warnings and reducing the mortality rate. The proposed classifier *CBCEC* significantly outperformed the baseline and state-of-the-art models. However, it needs to undertake multiple steps during the execution, as it demands significant computational resources compared to baseline classifiers. In the future, we aim to reduce the computational cost by integrating distributed learning mechanisms into our framework. Along with this, we would like to gather a sizable dataset to further improve our model's generalization.

## Data Availability

All data generated or analyzed during this study are included in this published article. It also available in- https://www.kaggle.com/datasets/andrewmvd/heart-failure-clinical-data.
